# Olivopontocerebellar Degeneration in a Young Adult Female: A Case Report of Early Onset and an Uncommon Course

**DOI:** 10.7759/cureus.69384

**Published:** 2024-09-14

**Authors:** Sanjaykanth B, Jasvant Ram Ananthasayanam, Sharmeela S, Arunkumar Mohanakrishnan, Karthik Krishna Ramakrishnan

**Affiliations:** 1 Radiodiagnosis, Saveetha Medical College and Hospital, Saveetha Institute of Medical and Technical Sciences (SIMATS) Saveetha University, Chennai, IND

**Keywords:** cerebellar atrophy, early-onset neurodegenerative disorder, magnetic resonance imaging (mri), olivopontocerebellar degeneration (opcd), pontine atrophy

## Abstract

Olivopontocerebellar degeneration (OPCD) primarily affects individuals in their mid to late adulthood, making its early onset in young adults, particularly postpartum women, a notable rarity. This case report describes OPCD in a 24-year-old female, underscoring the importance of considering neurodegenerative disorders in differential diagnoses even in younger patients. The unique presentation post childbirth adds to the sparse literature on the timing and triggers of neurodegenerative diseases in younger populations, especially in scenarios that might involve hormonal, vascular, or autoimmune shifts such as those occurring postpartum. The patient, a young female, presented with progressive cerebellar symptoms, including gait ataxia, characterized by unsteady walking, dysarthria, manifesting as slurred speech, and an intentional tremor noticeable during precise movements. Further clinical findings included nystagmus, involuntary eye movement, and dysmetria demonstrated in the finger-to-nose test. These symptoms progressively worsened after her first childbirth, emphasizing the progressive nature of the disease. The MRI findings were pivotal in diagnosing OPCD, revealing extensive cerebellar and pontine atrophy, particularly affecting the anterior lobe. The radiological features included significant thinning of the cerebellar folia, increased prominence of cerebellar fissures, and dilatation of the fourth ventricle. Based on these findings, the differential diagnosis included various other cerebellar ataxias, but the specific pattern of degeneration observed was indicative of OPCD. Therapeutically, the patient was managed with supportive physiotherapy and oral methylcobalamin supplementation aimed at slowing progression and alleviating symptoms. The outcome, while not curative, focuses on symptom management and improving quality of life. This case highlights the critical role of magnetic resonance imaging (MRI) in the early detection and diagnosis of OPCD, particularly in atypical patient populations such as young adults. It serves as a reminder to the medical community about the variability of presentation in neurodegenerative disorders and the need for vigilance in younger patients presenting with progressive neurological symptoms. Further, it emphasizes the importance of considering a comprehensive diagnostic approach, including detailed imaging studies when young patients present with atypical symptoms, to ensure accurate diagnosis and appropriate management.

## Introduction

Olivopontocerebellar atrophy (OPCA) is a neurodegenerative condition that primarily affects the pons, cerebellum, and olivary nuclei of the brain, leading to a range of motor and autonomic dysfunctions. Historically classified under the umbrella of multiple system atrophy (MSA), OPCA presents with complex diagnostic challenges due to its overlapping symptoms with other neurodegenerative disorders such as spinocerebellar ataxias (SCAs) and Parkinson's disease [1]. Olivopontocerebellar atrophy is distinct in its neuropathological features, which include significant atrophy in the cerebellum and pons, and it is characterized by cerebellar ataxia, dysarthria, and a variety of extrapyramidal symptoms. The clinical manifestation of OPCA often includes a combination of cerebellar signs and autonomic dysfunction, with some patients also displaying parkinsonian features such as rigidity and bradykinesia. This overlap of symptoms makes the clinical pathway for diagnosis particularly challenging, necessitating advanced imaging techniques and sometimes genetic testing to differentiate OPCA from other similar conditions [2]. Magnetic resonance imaging (MRI) remains the cornerstone of OPCA diagnosis, providing detailed visualization of the characteristic brainstem and cerebellar atrophy, and helping to rule out other causes of ataxia. As an entity, OPCA can present either as part of a hereditary ataxic condition or as a sporadic form, which is often linked to MSA. The sporadic form, in particular, is associated with alpha-synuclein pathology, classifying it among the alpha-synucleinopathies, which also include Parkinson's disease and Lewy body dementia. From a therapeutic perspective, management of OPCA remains symptomatic and supportive, with no current treatments available to halt or reverse the progression of the disease [3]. The focus is on managing symptoms such as ataxia, autonomic dysfunction, and Parkinsonism using a combination of pharmacological and non-pharmacological approaches [4].

This case of a young adult female presenting with OPCA post childbirth is particularly noteworthy due to the rarity of such early-onset cases and the potential implications for understanding the pathophysiological triggers involved, possibly linked to hormonal changes or autoimmune responses postpartum. It adds a significant piece to the complex puzzle of OPCA, challenging existing notions about the disease's etiology and progression, and underscores the need for ongoing research into its genetic and environmental triggers [5]. Neurodegenerative disorders in the postpartum period are rare, but there have been isolated case reports of conditions such as multiple sclerosis (MS), Guillain-Barré syndrome (GBS), and systemic lupus erythematosus (SLE)-related neuropsychiatric syndromes that may flare up during or after pregnancy. However, specific cases of OPCD or MSA related to pregnancy or the puerperium are exceedingly rare in the literature. Most reported postpartum neurodegenerative cases involve autoimmune disorders, where the immune reactivation post-pregnancy may lead to disease exacerbation or unmasking. This case contributes to the medical literature by highlighting the need for high clinical suspicion and comprehensive diagnostic approaches in atypical presentations of neurodegenerative diseases. Significant contributions from recent studies, including those by Rossi et al. (2018), who emphasized the need for clarity in the genetic nomenclature of recessive cerebellar ataxias, and Mormina et al. (2022), who refined MRI criteria for early OPCD diagnosis, highlight the evolving understanding of this complex disorder. These advancements in genetic research and neuroimaging have facilitated a more nuanced approach to diagnosis and management, aiding in the early detection and specific intervention strategies aimed at slowing disease progression and improving patient outcomes [6, 7].

## Case presentation

A 24-year-old primiparous female, two years postpartum, visited a neurology department with complaints of progressive neurological symptoms that began shortly after her first childbirth. Her primary symptoms included progressive imbalance, slurred speech, and difficulties with coordination. These symptoms appeared gradually but worsened noticeably over time, leading her to seek medical attention. The patient had no history of gestational hypertension, epilepsy, pyrexia, or postpartum hemorrhage, but the onset of symptoms during the puerperium raises questions about potential hormonal or autoimmune triggers. Upon neurological examination, the patient demonstrated significant signs of cerebellar dysfunction. Notably, she exhibited gait ataxia, characterized by uncoordinated and unsteady walking, and dysarthria, which manifested as slurred or slow speech. Additionally, an intentional tremor was evident during targeted movements, further complicating her motor skills. Other clinical findings included nystagmus, involuntary eye movement, and dysmetria on the finger-to-nose test, both indicative of substantial impairment in muscle movement and coordination control.

**Figure 1:**
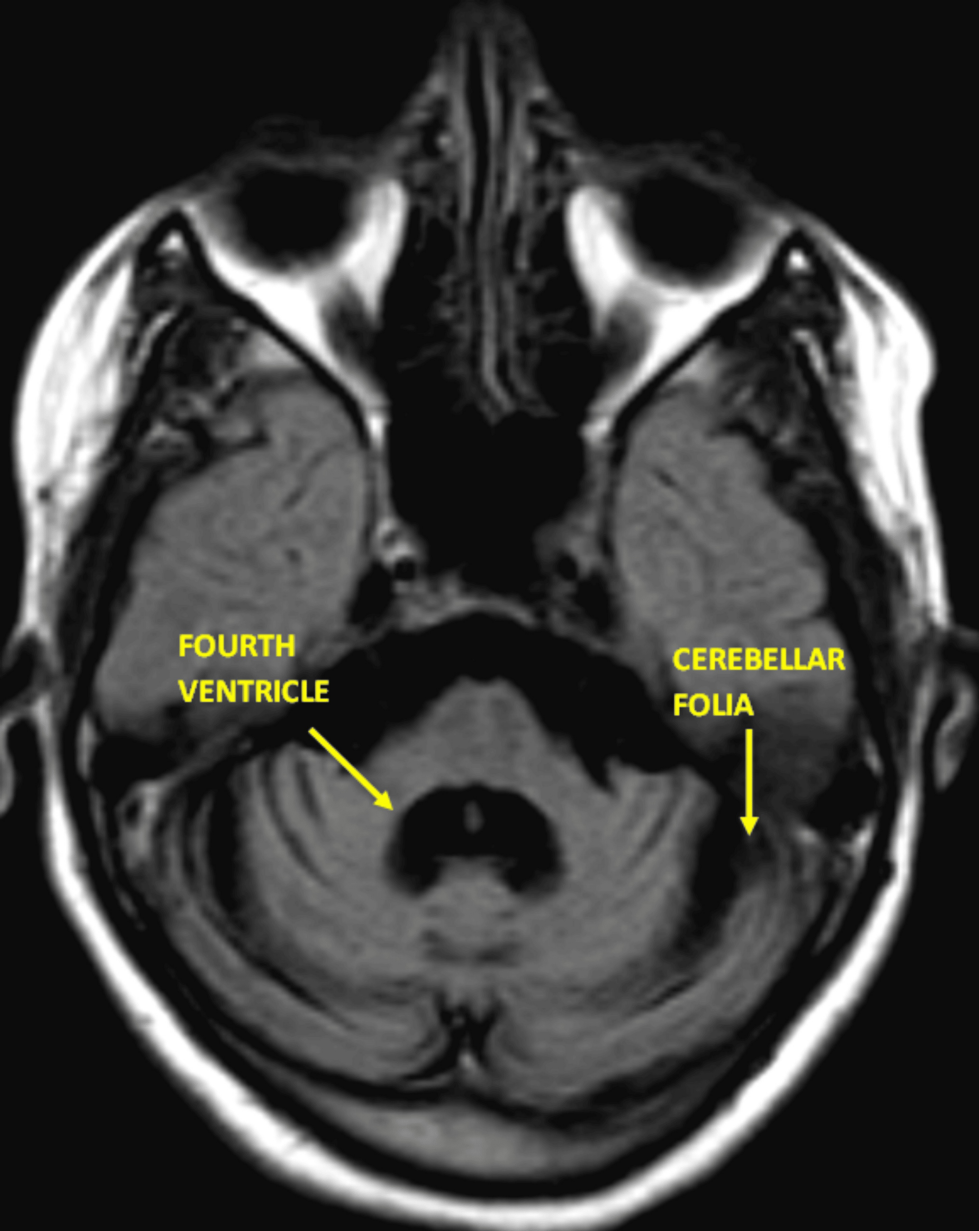
Axial view of the brain at the level of the posterior fossa; the cerebellum and brainstem are well visualized. There is notable atrophy of the cerebellum, particularly in the anterior lobe. The fourth ventricle appears dilated, which is consistent with the cerebellar atrophy.

**Figure 2:**
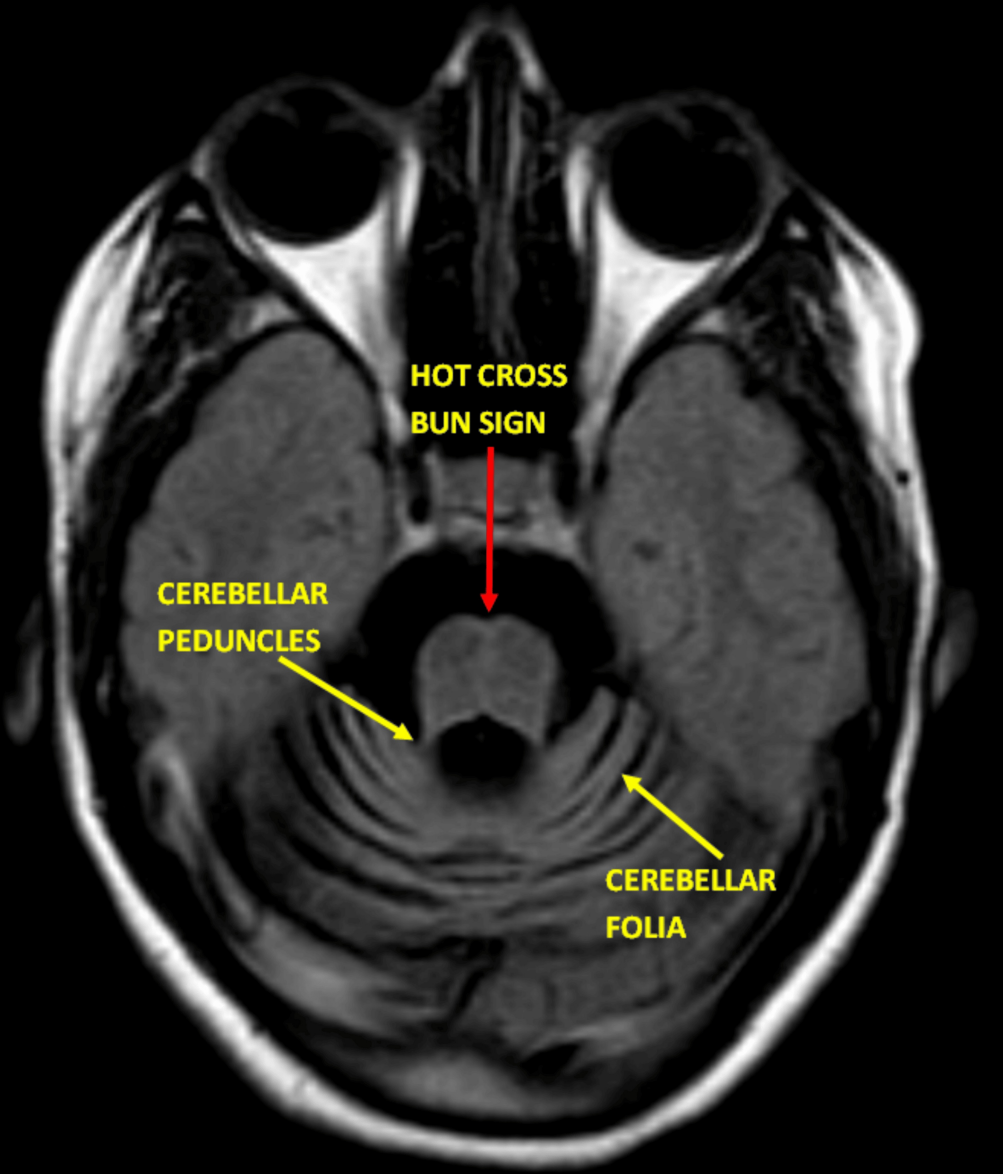
Axial FLAIR image shows atrophy involving the brainstem, cerebellum, and superior and middle cerebellar peduncles (yellow arrow). The cerebellar atrophy is evident with prominent cerebellar fissures and an enlarged fourth ventricle. Notably, the "hot cross bun" sign (red arrow) is apparent in this section across the pons, characterized by a cruciform hyperintensity that represents the degeneration of transverse pontocerebellar fibers and the pontine nuclei, a hallmark of olivopontocerebellar degeneration (OPCD). Additionally, there is a straightening of the pontomedullary angle, which is a further indicator of the atrophic changes in the brainstem. These features are highly suggestive of OPCD, reflecting the progressive neurodegenerative changes associated with this condition. The hyperintensities observed in the pons on this FLAIR image are indicative of gliosis, consistent with chronic neuronal loss. FLAIR: fluid-attenuated inversion recovery

Diagnostic imaging played a crucial role in elucidating the underlying pathology. An MRI of the brain revealed marked cerebellar atrophy, particularly affecting the anterior lobe (Figures 1-2). This atrophy was evidenced by significant thinning of the cerebellar folia and increased prominence of the cerebellar fissures, classic signs that underscore the progressive nature of cerebellar degeneration. Additionally, pontine atrophy was notably present (Figure 2). Such findings are not only diagnostic of OPCD but also help differentiate it from other similar neurodegenerative disorders.

**Figure 3:**
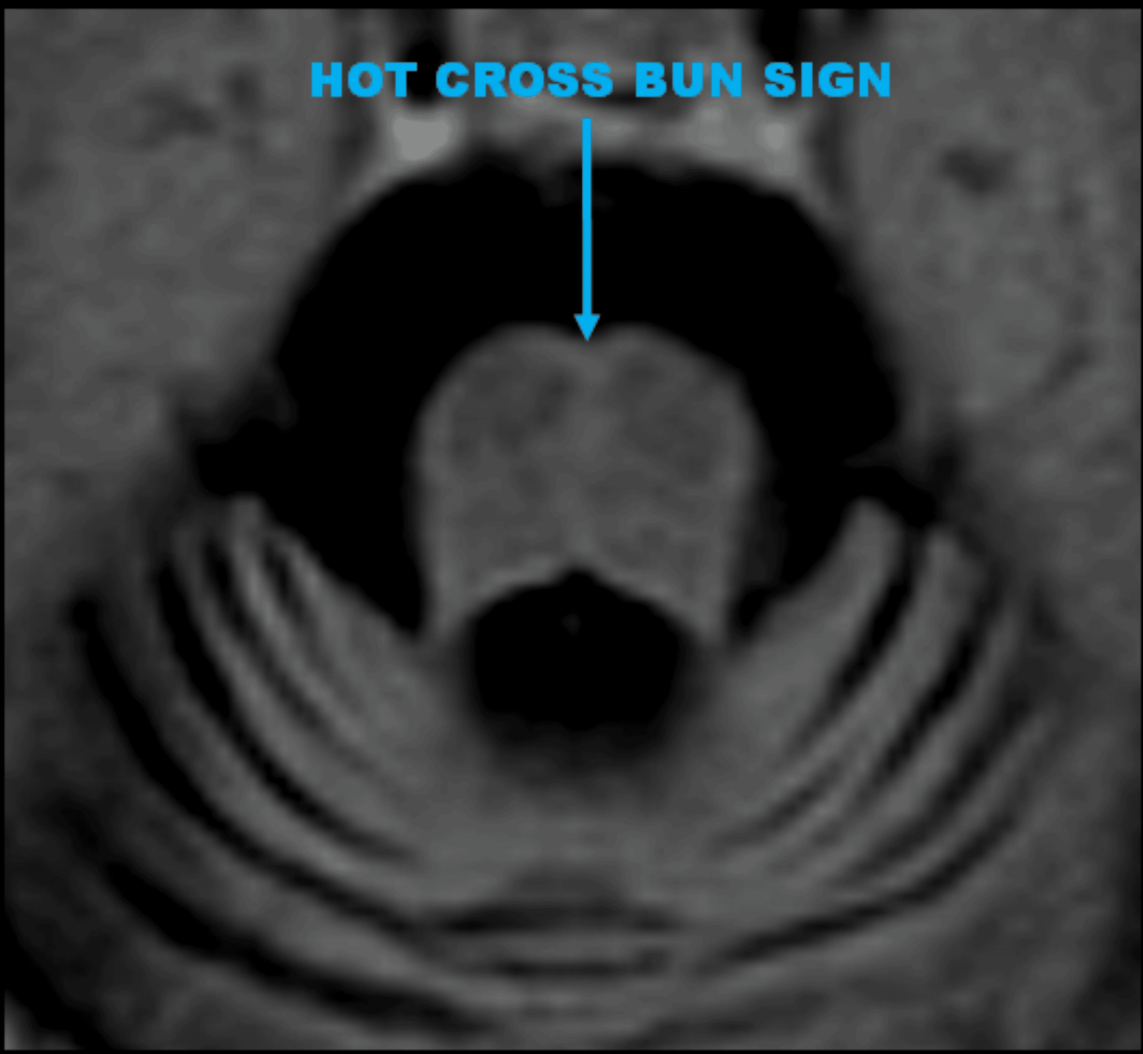
The axial image clearly demonstrates the "hot cross bun" sign, which is characteristic of olivopontocerebellar degeneration (OPCD). This sign represents selective degeneration of the transverse pontocerebellar tracts and the median pontine raphe nuclei, resulting in the cruciform appearance of hyperintensity within the pons. The image also shows significant atrophy of the brainstem, including the pons and middle cerebellar peduncles. The atrophy was accompanied by hyperintense signals in the pons, reflecting gliosis and chronic neuronal loss. Additionally, there was a straightening of the pontomedullary angle, further indicating the atrophic changes within the brainstem.

The radiological signature of OPCD also includes the presence of the "hot cross bun sign" on T2 fluid-attenuated inversion recovery (FLAIR)/T2-weighted images of the pons. The characteristic "hot cross bun" sign was observed, representing degeneration of the transverse pontocerebellar fibers and the pontine nuclei. This hallmark of OPCD provides critical diagnostic insight, as it distinguishes this condition from other cerebellar ataxias (Figure 3). This sign indicates degeneration of the pontocerebellar tracts, characterized by high-signal intensity, and is pivotal for distinguishing OPCD from conditions with similar presentations.

**Figure 4:**
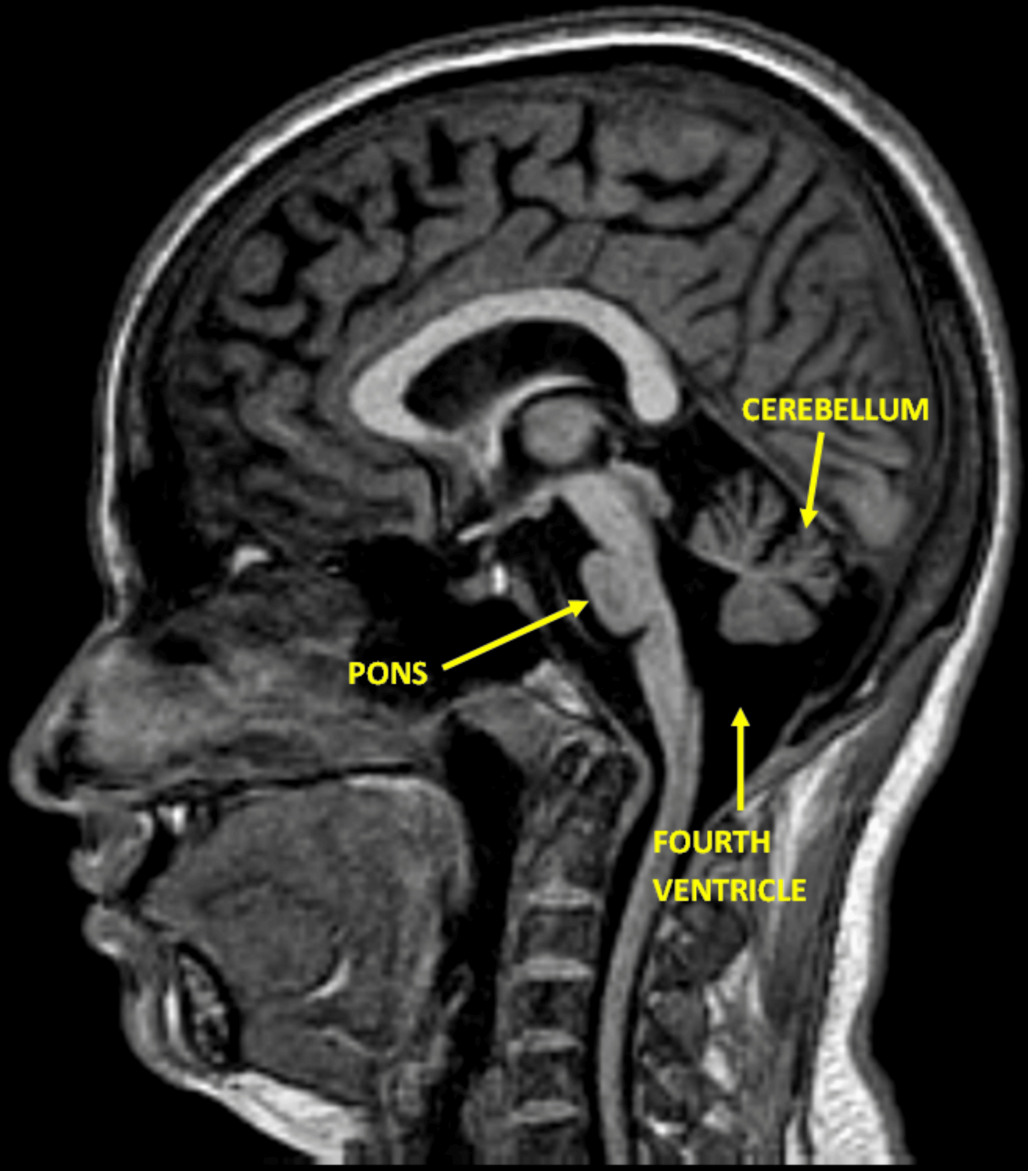
A sagittal T1-weighted image provides a side view of the brain, including the cerebellum, brainstem, and cerebral cortex. There is significant cerebellar atrophy, especially involving the vermis and the anterior lobe. The pons also appears atrophic, with a flattened ventral surface. The fourth ventricle is prominently dilated due to the atrophy. This image is crucial for assessing the extent of atrophy and the involvement of the brainstem structures.

**Figure 5:**
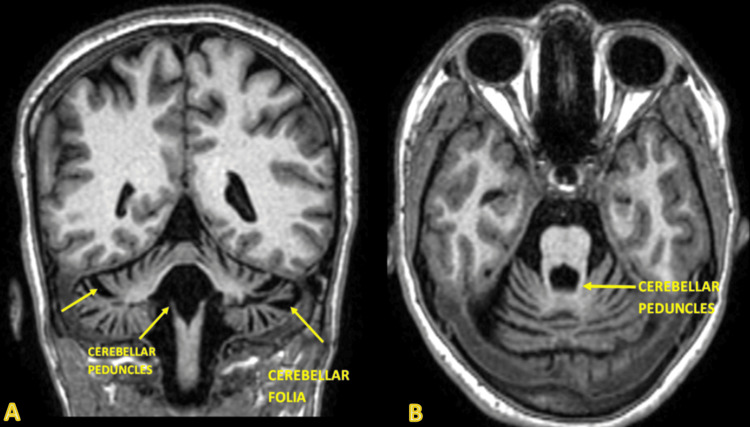
Coronal (A) and axial (B) T1-weighted image shows a front-to-back view of the brain. There is a clear atrophy of the cerebellar hemispheres with thinning of the cerebellar folia, cerebellar peduncles, and increased prominence of the cerebellar fissures. The fourth ventricle appears dilated. This image helps to assess the degree of cerebellar atrophy and its impact on surrounding structures.

Another critical observation was the notable dilatation of the fourth ventricle (Figures 4-5). This dilatation was a direct consequence of the atrophic changes within the cerebellum and pons indicated by the flattening of the ventral pons, thus leading to a compensatory expansion of the ventricular system. Moreover, the atrophy of the middle cerebellar peduncle was well identified, a finding that is consistent with the widespread cerebellar involvement seen in OPCD (Figure 5).

**Figure 6:**
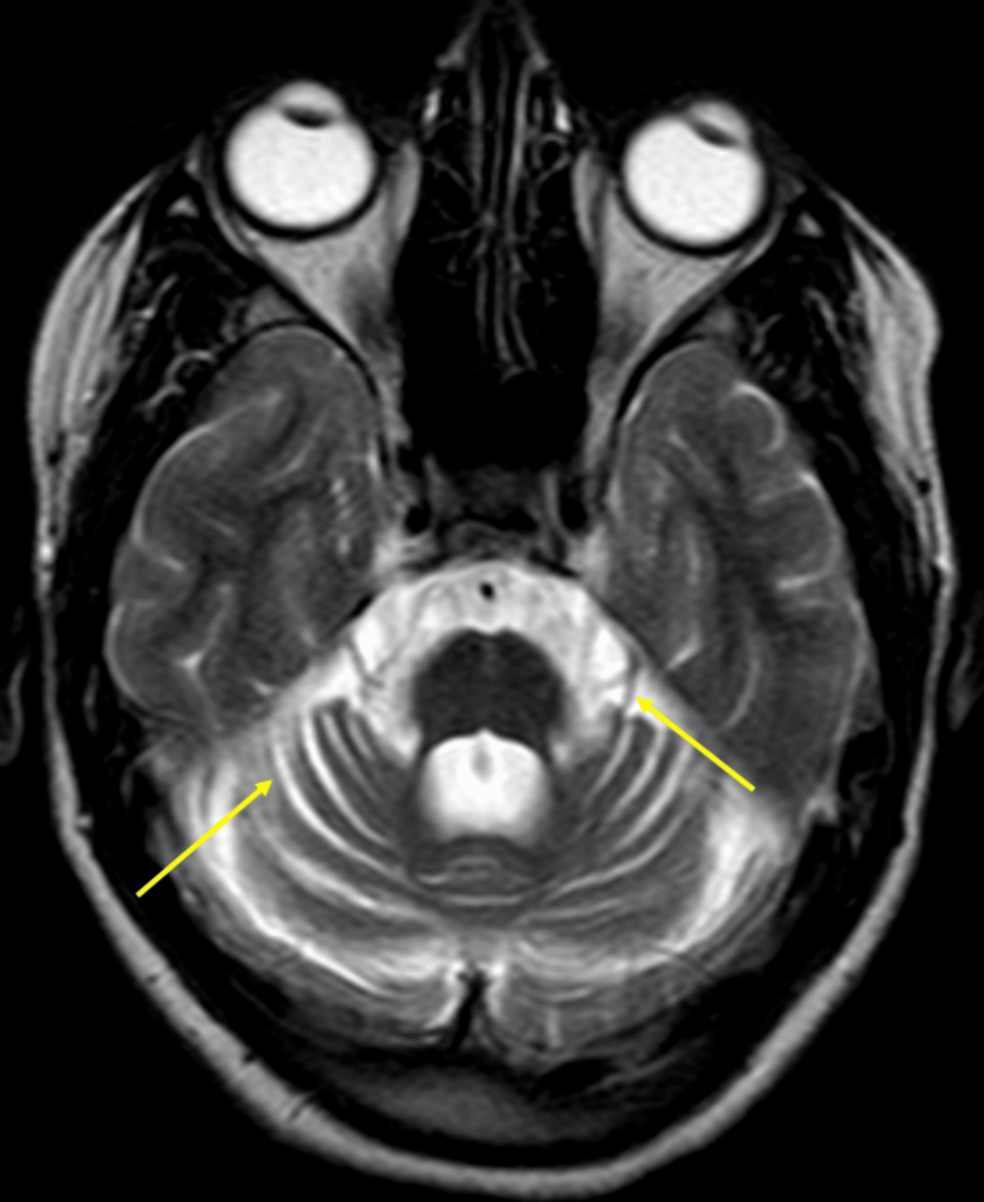
The axial T2-weighted image shows a higher signal in the cerebellar white matter and the brainstem, reflecting gliosis or degeneration. The cerebellar atrophy is again evident with dilatation of the fourth ventricle and prominent cerebellar fissures. The T2-weighted image is useful for visualizing the extent of white matter changes and atrophy.

**Figure 7:**
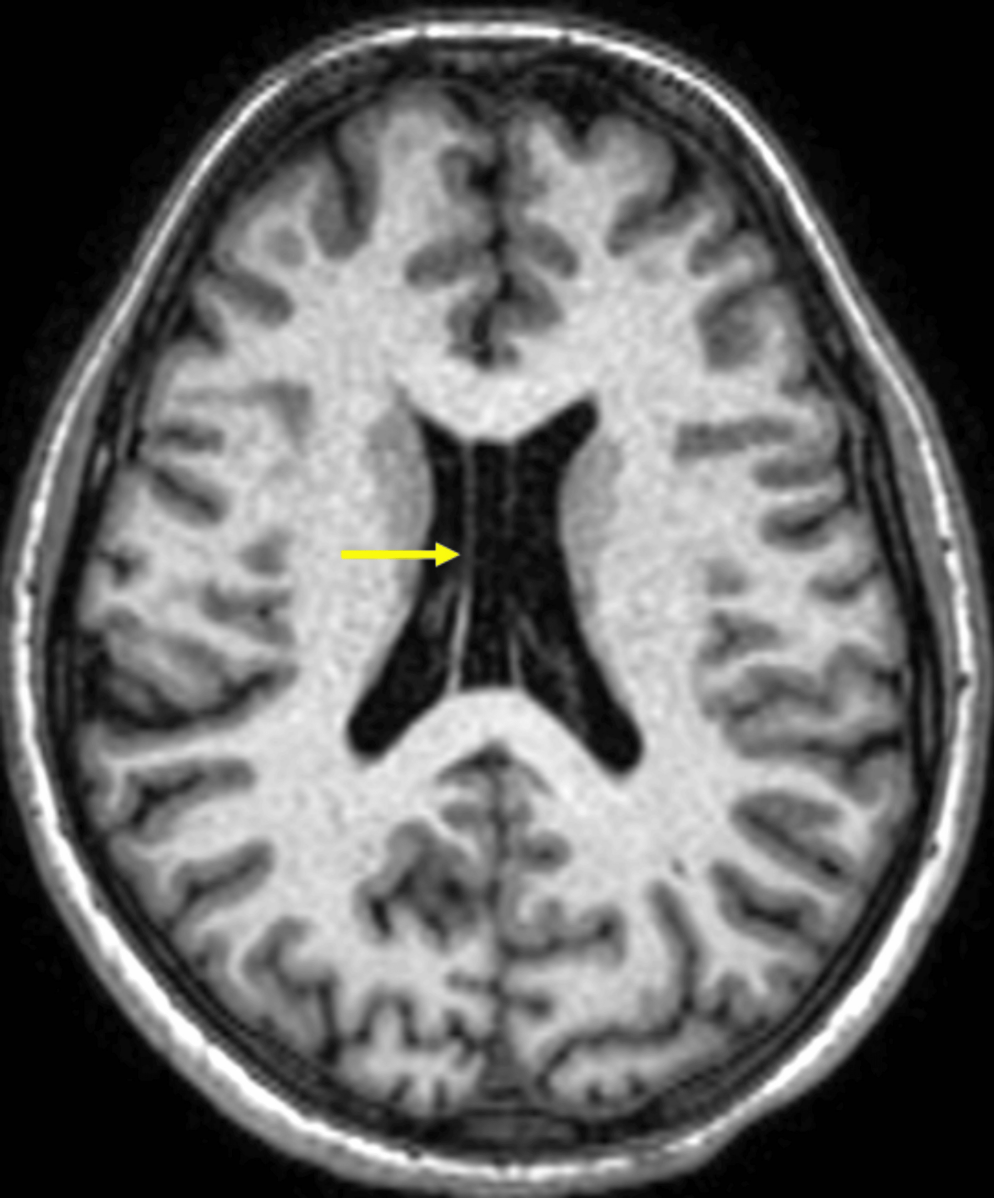
Axial section shows the cerebral hemispheres, cortex, and lateral ventricles. The cortex appears relatively preserved, but the lateral ventricles are slightly enlarged, which could be a compensatory change due to the cerebellar atrophy. The image provides a reference for normal brain anatomy in contrast to the affected cerebellum and brainstem in other images. Note is also made of cavum septum pellucidum et cavum vergae (arrow).

T2-weighted images further revealed hyperintensities within the pons, suggesting the presence of neuronal loss and gliosis, a reactive change often seen in chronic degenerative processes (Figure 6). The diagnostic process was guided by the identification of specific radiological features on MRI, crucial for an accurate diagnosis of OPCD. Among these, the most notable sign is the cerebellar atrophy, particularly involving the anterior lobe. Detailed imaging on sagittal T1-weighted views showed marked atrophy of the cerebellar vermis, with the flattened appearance of the ventral pons providing additional diagnostic confirmation (Figures 6-7).

The differential diagnosis for this patient was challenging, given the overlap of her symptoms with other neurodegenerative disorders. Conditions considered included spinocerebellar ataxias, multiple system atrophy-cerebellar subtype (MSA-C), Friedreich’s ataxia, late-onset Tay-Sachs disease, and idiopathic late-onset cerebellar ataxia. Each of these conditions shares similar radiological features with olivopontocerebellar degeneration, such as cerebellar and brainstem atrophy. However, the specific atrophy patterns and the progression of symptoms were most consistent with OPCD, leading to a final diagnosis. The management of this patient remains supportive and symptomatic, with a focus on physiotherapy to improve motor coordination and oral methylcobalamin supplementation to support nerve function. Given the progressive nature of OPCD and the absence of curative treatments, the prognosis is guarded with an expected gradual worsening of symptoms over time. This case underscores the importance of early and accurate imaging in diagnosing complex neurodegenerative disorders and highlights the unique challenges of managing such conditions in younger patients, particularly in postpartum women.

## Discussion

Olivopontocerebellar degeneration, a form of MSA, is a progressive neurodegenerative disorder that typically presents in middle-aged to older adults [7]. The presentation of OPCD in a young postpartum female, as detailed in this case report, is exceptionally rare and highlights the necessity of considering OPCD in younger patients, especially when clinical symptoms are atypical for age. The early onset of OPCD symptoms occurred during the puerperium period, suggesting a possible link to postpartum hormonal or vascular changes. However, there were no significant signs during pregnancy, indicating this as a likely idiopathic and isolated phenomenon. This raises questions about potential pathophysiological mechanisms linked to hormonal or autoimmune responses that may be triggered by the postpartum state. Neurodegenerative conditions such as OPCD are typically associated with genetic predispositions and environmental factors. However, the timing of symptom onset in this case suggests a possible role of hormonal fluctuations or vascular changes that occur during and after pregnancy, which may have exacerbated or unmasked an underlying neurodegenerative process [8]. This case, with symptom onset shortly after childbirth, raises the possibility of postpartum-related triggers for OPCD. While no specific immunological markers have been identified linking OPCD to pregnancy-related changes, the postpartum period is known for immune reactivation, which has been associated with flare-ups in other neurodegenerative and autoimmune disorders. The absence of identified autoimmune markers in OPCD emphasizes the need for further research to explore the role of hormonal and immune factors in triggering early disease onset in postpartum patients. While the direct pathophysiological link remains unclear, hormonal changes during pregnancy and the postpartum period, such as fluctuations in estrogen and progesterone levels, may influence neuroinflammatory pathways. Additionally, immune reactivation and vascular changes could exacerbate or unmask underlying neurodegenerative processes [7, 8].

Olivopontocerebellar degeneration, a form of MSA, is characterized by progressive atrophy of the pons, cerebellum, and inferior olives. Olivopontocerebellar degeneration itself is a heterogeneous condition with several subtypes, each distinguished by specific clinical and pathological features. The major subtypes include hereditary OPCD, sporadic OPCD, and OPCD associated with MSA-C. Hereditary OPCD is often linked to autosomal dominant inheritance patterns and may present as part of a broader spectrum of neurodegenerative disorders such as SCAs. These hereditary forms of OPCD are typically associated with specific gene mutations, such as those seen in SCA types 1, 2, 3, and 6, where the degeneration predominantly affects the cerebellum and brainstem, leading to symptoms like gait ataxia, dysarthria, and intention tremor [9-11]. In contrast, sporadic OPCD, which is not linked to a familial history, often overlaps with MSA-C [10, 11]. Multiple system atrophy-cerebellar subtype is characterized by prominent autonomic dysfunctions alongside cerebellar and pyramidal symptoms, distinguishing it from the more isolated cerebellar ataxias seen in hereditary forms. Each subtype of OPCD presents with a varying pattern of neurodegeneration, which can be visualized through advanced neuroimaging techniques. For instance, hereditary forms may show more isolated cerebellar involvement, while MSA-C is typically associated with widespread neurodegeneration, including pontine and olivary structures, and often shows the characteristic "hot cross bun" sign on MRI [10].

Magnetic resonance imaging played a pivotal role in diagnosing OPCD in this patient. The neuroimaging findings of significant cerebellar and pontine atrophy, particularly involving the anterior lobe of the cerebellum, are consistent with the classic presentation of OPCD [12]. The "trident" or "cross" sign observed on T2-weighted images is characteristic of OPCD and serves as a crucial diagnostic marker, helping to differentiate it from other forms of cerebellar ataxia, such as SCAs and idiopathic late-onset cerebellar ataxia. The neuroimaging findings in OPCD are critical not only for diagnosis but also for monitoring disease progression [13]. In this patient, the presence of hyperintensities within the pons on T2-weighted images suggests ongoing neuronal loss and gliosis, which are hallmark features of chronic neurodegenerative processes [14]. The differential diagnosis for this patient was extensive, including conditions like SCAs, MSA-C, and Friedreich’s ataxia. However, the specific pattern of atrophy and the clinical progression observed in this case were most consistent with OPCD [15]. Further research is needed to explore these potential mechanisms in early-onset OPCD.

## Conclusions

In conclusion, this case of early-onset OPCD in a postpartum female underscores the importance of considering neurodegenerative disorders even in younger patients, particularly when symptoms arise in the postpartum period. The unusual timing of onset following childbirth suggests a potential role for hormonal or autoimmune triggers, emphasizing the need for further research into the pathophysiological mechanisms that may accelerate neurodegeneration in such contexts. The diagnostic process, anchored by detailed MRI findings, was critical in identifying the characteristic cerebellar and pontine atrophy specific to OPCD, highlighting MRI’s essential role in differentiating this condition from other cerebellar ataxias. Although treatment remains largely supportive, focusing on symptom management through physiotherapy and pharmacological interventions like methylcobalamin, this case reinforces the necessity of a multidisciplinary approach to care. It also highlights the progressive nature of OPCD and the importance of early detection in improving patient outcomes. This case contributes to the expanding knowledge of OPCD, challenging the traditional age boundaries of neurodegenerative diseases, and underscores the need for vigilance in recognizing and diagnosing such conditions in younger, atypical patient populations.
